# Observation of dielectric universalities in albumin, cytochrome C and *Shewanella oneidensis* MR-1 extracellular matrix

**DOI:** 10.1038/s41598-017-15693-y

**Published:** 2017-11-16

**Authors:** K. A. Motovilov, M. Savinov, E. S. Zhukova, A. A. Pronin, Z. V. Gagkaeva, V. Grinenko, K. V. Sidoruk, T. A. Voeikova, P. Yu. Barzilovich, A. K. Grebenko, S. V. Lisovskii, V. I. Torgashev, P. Bednyakov, J. Pokorný, M. Dressel, B. P. Gorshunov

**Affiliations:** 1 Moscow Institute of Physics and Technology, Dolgoprudny, Moscow Region Russia; 2 Institute of Physics AS CR, Praha 8, Czech Republic; 3 A.M. Prokhorov General Physics Institute, RAS, Moscow, Russia; 4 Institute for Metallic Materials, IFW Dresden, Dresden, Germany; 5 1. Physikalisches Institut, Universität Stuttgart, Stuttgart, Germany; 6 Scientific Center of Russian Federation Research Institute for Genetics and Selection of Industrial Microorganisms, Moscow, Russia; 7 Southern Federal University, Rostov-on-Don, Russia; 8 Moscow Institute of Physics and Technology, Institutsky lane 9, Dolgoprudny, Moscow, 141701 Russia

## Abstract

The electrodynamics of metals is well understood within the Drude conductivity model; properties of insulators and semiconductors are governed by a gap in the electronic states. But there is a great variety of disordered materials that do not fall in these categories and still respond to external field in an amazingly uniform manner. At radiofrequencies delocalized charges yield a frequency-independent conductivity *σ*_1_(*ν*) whose magnitude exponentially decreases while cooling. With increasing frequency, dispersionless conductivity starts to reveal a power-law dependence *σ*_1_(*ν*)∝*ν* ^*s*^ with *s* < 1 caused by hopping charge carriers. At low temperatures, such Universal Dielectric Response can cross over to another universal regime with nearly constant loss ε″∝σ_1_/ν = const. The powerful research potential based on such universalities is widely used in condensed matter physics. Here we study the broad-band (1–10^12^ Hz) dielectric response of *Shewanella oneidensis* MR-1 extracellular matrix, cytochrome C and serum albumin. Applying concepts of condensed matter physics, we identify transport mechanisms and a number of energy, time, frequency, spatial and temperature scales in these biological objects, which can provide us with deeper insight into the protein dynamics.

## Introduction

It is well established that the electrodynamic behavior of the vast majority of amorphous semiconductors, ionic conductors, glasses, etc., is summarized in the universal dielectric response (UDR), i. e. phenomenological description suggested by Jonscher 40 years ago. At elevated temperatures the low-frequency (hertz to megahertz) charge transfer is governed by quasi-free carriers, following the Drude conductivity model^1,2^ with a frequency independent real part of the conductivity *σ*_1_. In the terahertz spectral range and above hopping type of conductivity becomes dominant. That is described by Mott frequency dependence *σ*_1_~*ν* ^*s*^ with the value of hopping exponent *s* ≈ 0.8^3^. Upon cooling, the charge carriers become increasingly localized, i.e. the Drude-type response exponentially freezes out, and the hopping regime extends to lower frequencies. The described temperature-frequency behaviors are perfectly reproduced by phenomenological Jonscher’s equation^4–8^:

1 $${\sigma }_{1}(\nu )={\sigma }_{dc}[1+{(\frac{\nu }{{\nu }_{cr}})}^{s}]$$

where *σ*_dc_ is the dc condutivity and *ν*_cr_ is the frequency of crossover between the Drude-like and Mott-like regimes; it is usually taken as the frequency at which σ_1_(ν_cr_) = 2σ_dc_^9^.

In the low-temperature and high-frequency limit, the Jonscher regime often transforms into another, also universal spectral response, called nearly constant loss (NCL), since here the conductivity is proportional to frequency implying frequency-independent loss *ε*″ = const. In many cases the experimental data on dc/ac conductivity and dielectric permittivity of disordered conductors obtained at different frequencies and temperatures can be merged on a single master curve thus signifying the frequency-temperature superposition principle^10,11^. Such universal scalings are revealed by plotting the reduced conductivity or permittivity versus reduced frequency^5,6,9,10^:

2 $$\frac{{\sigma }_{1}(\nu )}{{\sigma }_{dc}}=F\,(\frac{\nu }{{\sigma }_{dc}T});\frac{\sigma (\nu )}{{\sigma }_{dc}}={F}_{1}(\frac{{\rm{\Delta }}\varepsilon }{{\sigma }_{dc}}\nu );\frac{\varepsilon \text{'}(\nu )-{\varepsilon }_{\inf }}{{\rm{\Delta }}\varepsilon }={F}_{2}(\frac{{\rm{\Delta }}\varepsilon }{{\sigma }_{dc}}\nu );$$

Here *F*, *F*_1_ and *F*_2_ are scaling functions, *ε*‘ is the real permittivity, Δ*ε* is the dielectric strength of hopping transport and *ε*_inf_ is the dielectric contribution from high frequency excitations. The above dielectric universalities were revealed on the basis of decades-long spectroscopic investigations on vast variety of disordered and amorphous materials and they have been helping physicists in analyzing the general regularities in dynamical behaviors in seemingly quite different systems thus giving a deeper understanding of their intrinsic properties.

We have applied broad-band dielectric spectroscopy to measure the spectra of ac conductivity and dielectric permittivity of three different materials of biological origin. Two of them, bovine serum albumin (BSA) and bovine heart cytochrome C (CytC), represent reference materials with well studied properties and structure^12,13^ and, at the same time, have rather different relation to natural electronic conductivity in biosystems. CytC is a part of oxidative phosphorylation system in mitochondria and bacteria^14^. It represents one of many links within chain of electron transfer reactions and contains specific low-molecular structure, derivative of porphyrin, called cytochrome. Various cytochromes, iron-sulfur and copper clusters are presently regarded as a characteristic structural feature of the proteins responsible for electron transfer in biosystems^15^. These prosthetic groups govern redox properties of the whole macromolecule and form the local states for electrons which “hop” or tunnel from one local state to another during chemical redox reactions within proteins^12,15,16^.

BSA represents another type of proteins. It does not contain any prosthetic group and, as far as it is currently known, has no direct relation to electron transfer processes. The original function of serum albumin is a transfer of various hydrophobic substances and maintaining of oncotic pressure within blood plasma^13^.

CytC and BSA are classical reference standardized materials in various studies devoted to peculiarities of the proteins since they have well studied structure, physical and chemical properties. Not less important is that they are relatively cheap and commercially available in a stable and controllable state.

The third material studied here is extracellular matrix and filaments (EMF) of electrogenic bacteria *Shewanella oneidensis* MR-1. The ability of some microorganisms to produce technologically notable electrical currents in so-called biological fuel cells is a prominent hallmark of the surveys devoted to the search for alternative renewable energy sources since the beginning of 1990-s^17^. Several interesting corresponding species were found and *S. oneidensis*, being energy omnivore, became one of the most popular in the field. In 2000–2010 s, it was demonstrated that at least several species of electrogenic bacteria, including *S. oneidensis*, utilize certain outer cell structures with filamentous spatial organization to transfer electrons from cell to oxidizing substrate (anode, minerals, other bacterial cells, etc.)^18^. Intensive studies of conductivity within single separate filaments of bacterial cell were performed by the group of M.El-Naggar^19–21^. They demonstrated the presence of the signs of p-type semiconducting behavior within some filaments^22^, signatures of periplasmic origin of filaments^23,24^ and particularly underlined that not all of the filaments of *S. oneidensis* are conductive (see supplementary materials in^19^). At the same time, their methodology suffered from the lack of wide range temperature dependent measurements of conductivity, absence of analysis of ions and water concentration in the studied objects and application of aldehyde-based chemical fixation of the samples. Importantly, some of these factors can significantly influence the values and mechanisms of conductivity. Unlike M.El-Naggar *et al*., we did not separate pure fraction of the filaments from the outer cell matrix. Our material contained different filaments and other components of the extracellular matrix (Supplementary Fig. 1 is given as an example). In our study we did not have a purpose to examine the conductivity just of filaments of certain type. Our goal was to take a look at a generalized picture, where all types of conductivities attributable to species’ extracellular structures had to show up.

## Results and Discussion

We used dielectric spectroscopy techniques with the frequency range of the probing radiation as broad as 1 to 10^12^ Hz. The temperature range of performed measurements was 10–310 K. CytC and BSA were lyophilized powders supplied by SigmaAldrich and Amresco, respectively. Water content in all samples was controlled by thermogravimetry technique (Supplementary Fig. 2). Concentrations of metals in the samples were measured by means of mass-spectrometry (Supplementary Table 1). The presence of multiheme cytochromes in EMF was controlled with EPR analysis (Supplementary Fig. 3) since this technique was already broadly applied in the field^25–27^. Also, we applied circular dichroism technique to detect secondary protein structure distribution within samples (Supplementary Fig. 4). In addition to spectroscopic, we have performed measurements of the dc conductivity of EMF at 270–310 K and of the heat capacity of all three materials. We note that we did not detect any noticeable hysteresis effects in electric, dielectric, thermodynamic or magnetic measurements of the three materials. The details on samples’ preparation, characterization and measurements techniques are given in the section Materials and Methods.

### Dielectric spectroscopy of BSA, CytC and EMF

**Figure 1:**
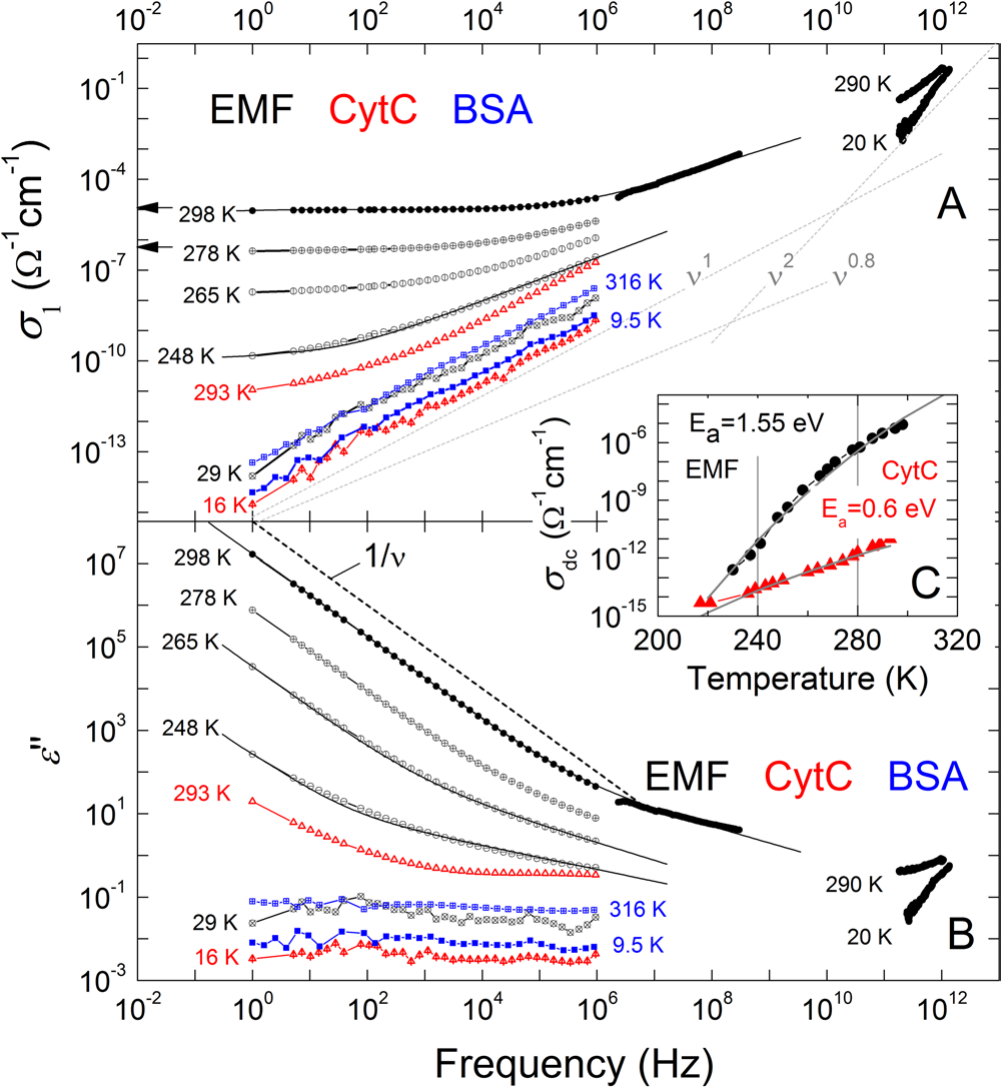
Broad-band spectra of real part of conductivity (**A**) and imaginary part of dielectric permittivity (**B**) of EMF (black circles), CytC (red triangles) and BSA (blue squares) measured at different temperatures. Solid lines represent least-square fitting results based on expression (1) in the text. Dotted lines in frame (**A**) indicate the *σ*_1_∝ ν^1^ dependence that corresponds to the nearly constant loss behavior *ε*″(*ν*) = const. The *σ*_1_∝ *ν* ^0.8^ dependence is typical for hopping transport. Flattening towards low frequencies of the room temperature *σ*_1_(*ν*) spectra and corresponding increase in the *ε*″(*ν*) spectra [*ε*″(*ν*) ∝ *ν* ^−1^ as shown with the dashed line] of CytC are due to finite dc conductivity, as discussed in the text. The ν^2^ behavior corresponds to the low-frequency “tail” of a Lorentzian-like absorption resonance in the terahertz range (see also Supplementary Fig. 5). The black arrows correspond to dc-measurement of EMF conducted by means of four-probe technique at 278 and 298 K. The inset (**C**) demonstrates the temperature dependence of the dc conductivity of EMF and CytC samples. Solid lines correspond to activated behaviors as discussed in the text. Water contents in samples according to thermogravimetry measurements: EMF (~30%), CytC (~13%), BSA (~11%).

Figure 1A shows the spectra of conductivity *σ*_1_(*ν*) of EMF, BSA and CytC. At room temperature, the conductivity of EMF is dispersionless in a wide frequency range (over five decades starting with 1 Hz). It indicates the Drude-type response^1,2^ of delocalized charge carriers, also seen in the dispersion of imaginary part of permittivity *ε*″ ∝ *σ*_1_(*ν*)/*ν* ∝ 1/*ν* (Fig. 1B). Above ≈1 MHz, the *σ*_1_(*ν*) behavior of EMF crosses over to a hopping-like^3^ frequency dependence *σ*_1_ ∝ *ν* ^*s*^ with *s* ≈ 0.6. While cooling, the Drude component exponentially weakens in magnitude and completely freezes out below T = 240–245 K. The observed pattern is perfectly described by the Jonscher expression (1). The same behavior is observed at room temperature in the case of CytC sample: at the lowest frequencies the Drude-type dependence flattens off and the *σ*_1_(*ν*) spectrum gradually transforms to the *σ*_1_ ∝ *ν* ^*s*^ behavior at temperatures below 275 K. The BSA sample at all temperatures demonstrates only a *σ*_1_ ∝ *ν* ^*s*^ behavior with *s* ≈ 0.96. Processing (using expression 1) the obtained temperature dependences of *σ*_dc_ reveals an activated character with activation energies *E*_a_ ≈ 1.55 eV for EMF and *E*_a_ ≈ 0.6 eV for CytC (Fig. 1C). The ν^2^ behavior corresponds to the low-frequency “tail” of a Lorentzian-like absorption resonance in the terahertz range (see also Supplementary Fig. 5).

### Scaling functions for EMF and CytC

**Figure 2:**
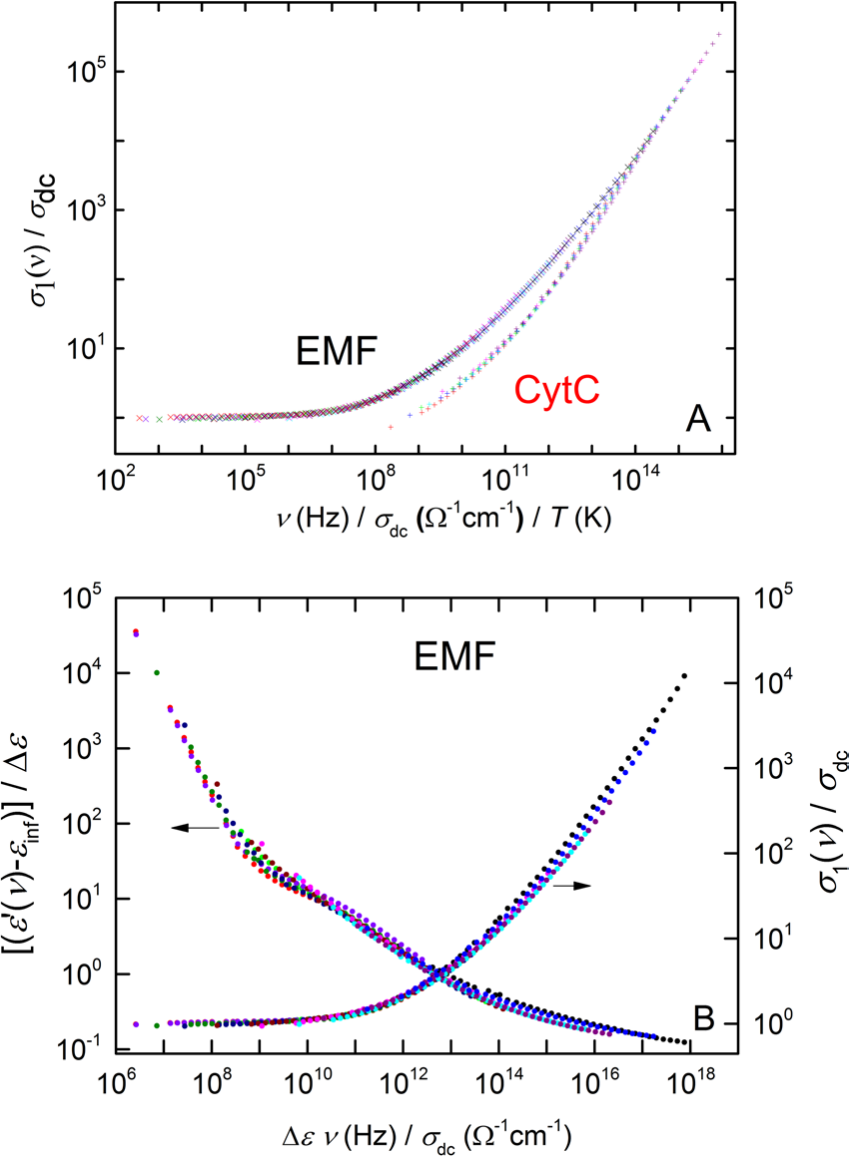
(**A**) Scaling of the ac conductivity of EMF (different colors correspond to temperatures 298, 296, 290, 283, 278, 274, 265, 258, 255, 248 and 245 K) and СytC (temperatures 293, 289, 286, 283, 280 and 278 K) samples according to $\frac{{\sigma }_{1}(\nu )}{{\sigma }_{dc}}=F(\frac{\nu }{{\sigma }_{dc}T});$ (**B**) scaling of ac conductivity of EMF according to $\frac{\sigma (\nu )}{{\sigma }_{dc}}={F}_{1}(\frac{{\rm{\Delta }}\varepsilon }{{\sigma }_{dc}}\nu )$ and $\frac{\varepsilon \text{'}(\nu )-{\varepsilon }_{\inf }}{{\rm{\Delta }}\varepsilon }={F}_{2}(\frac{{\rm{\Delta }}\varepsilon }{{\sigma }_{dc}}\nu )$.

To demonstrate that the high temperature charge dynamics is governed by the fundamental frequency-temperature superposition principle^10,28^, we show in the Fig. 2 that all three scaling relations (2) are perfectly satisfied in the cases of EMF and CytC samples at temperatures, where noticeable dispersionless free-carrier conductivity is detected. The distinction between the scaling functions *F* obtained for EMF and CytC (see Fig. 2A where the groups of “collapsed” data points related to EMF and CytC do not fully coincide) can be regarded as a signal of a difference between types of free carriers. Unfortunately, having this data is not sufficient to testify exactly what type of carriers defines the Drude-type conductivity in each material.

### Nearly constant loss (NCL)

**Figure 3:**
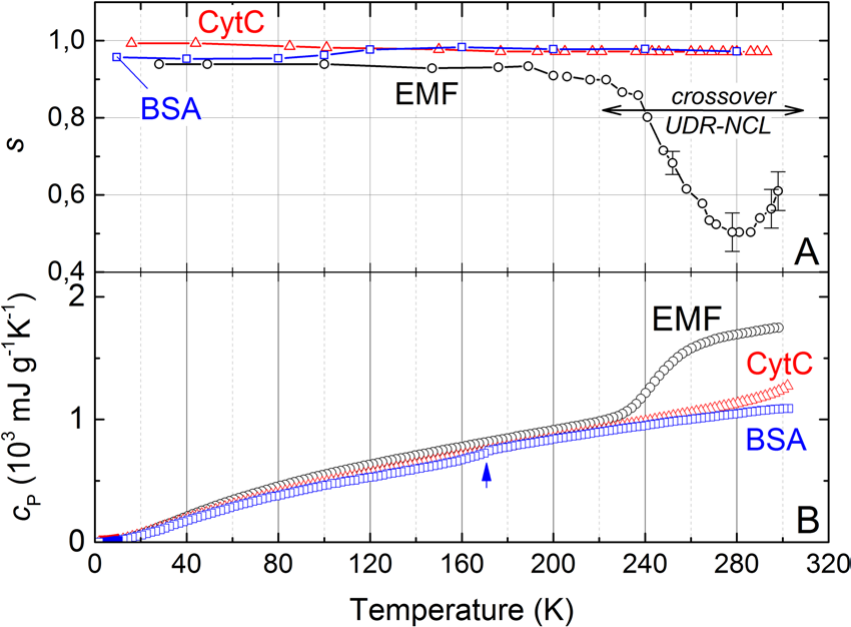
(**A**) Temperature dependence of the hopping exponent *s* in the equation (1) for EMF (black circles), CytC (red triangles) and BSA (blue squares) samples. UDR-NCL crossover in EMF is indicated by two-edged arrow. (**B**) Temperature dependence of heat capacity *c*_P_ for EMF (black circles), CytC (red triangles) and BSA (blue squares). The feature at the BSA data marked by an arrow can be caused by water molecules localized in pores as is observed in lysozyme^65^. Water contents in samples according to thermogravimetry measurements: EMF (~30%), CytC (~13%), BSA (~11%).

During cooling down from room temperature, pronounced changes of the hopping exponent *s* are observed for the EMF sample. It passes through a broad minimum and below *T* = 160–170 K saturates to a value *s* ≥ 0.94 (Fig. 3A). We associate this low-temperature response with the second dielectric NCL universality characterized by *ε*″(*ν*)∼*σ*_1_/*ν* = const^11,29,30^. We consider the temperatures 200–300 K as an interval, where the UDR-NCL crossover takes place in EMF. No significant variations of the hopping exponent are seen for BSA and CytC in the whole temperature interval. This observation testifies the NCL response without noticeable admixture of the hopping regime even at higher temperatures. Activation energies of the NCL process in samples are collected in Table 1. According to the qualitative picture for the protein dynamics proposed in^31^, the values of these energies (5–16 meV) may correspond to conformational fluctuations in the protein groups with amplitudes of 0.3–0.5 Å. Additional experimental studies and theoretical analysis are needed before more detailed microscopic picture for molecular dynamics could be built.

**Table 1:** Parameters of EMF, CytC and BSA: Debye temperature *Θ*_D_, Debye frequency *ν*_D_ = k_B_ *θ*_D_/h (k_B_ – Boltzmann constant, h – Planck constant), boson peak frequency *ν*_BP_ , boson peak correlation length *ξ*_BP_ , activation energies of the UDR and the NCL regimes *E*_a_(UDR) and *E*_a_(UDR), respectively, dipole moment.

Parameter	EMF	CytC	BSA
*Θ*_D_, K	110 ± 20	100 ± 20	90 ± 20
*ν*_D_, THz	2.3 ± 0.4	2.08 ± 0.4	1.88 ± 0.4
*ν*_BP_, GHz	750 ± 200	630 ± 150	540 ± 130
*ξ*_*BP*_, nm	2.7 ± 0.6	3.2 ± 0.6	3.8 ± 0.7
*E*_a_(UDR), meV	1550 ± 300	600 ± 130	—
*E*_a_(NCL), meV	15.5 ± 3	5.2 ± 0.5	9.5 ± 0.8
Approximate dimensions, Å	—	25·25·37^66^	140·40·40^67^
Dipole moment, Debye	—	~320^68^	~400^69^
*c*_*P*_-*T* freezing anomaly (Fig. 2A)	Yes	No	No
UDR-NCL crossover (Fig. 2B)	Yes	No	No
Free charge carriers	Yes	Yes	No
Hemes in structure	Yes	Yes	No
Quinones and flavins in structure	Yes	No	No
UDR regime observed	Yes	Yes	No
NCL regime observed	Yes	Yes	Yes
UDR-NCL-crossover	Yes	Yes	No
Boson peak observed in *c*_*P*_(*T*)	Yes	Yes	Yes
Boson peak observed in THz spectra	Yes	No	No

The indicated uncertainties correspond to the ranges of the data that provide satisfactory description of the original experimentally obtained material. Water contents in samples according to thermogravimetry measurements: EMF (~30%), CytC (~13%), BSA (~11%).

Precise microscopic origin of the caging potential, that can cause^28,32^ the NCL-type molecular response, may originate from the dynamics of polar protein molecules or molecular complexes and from caged water molecules dynamics^33,34^.

### Heat capacity, terahertz spectral response and signatures of loosely bound water

Around 235–255 K, a knee-like feature is seen in the specific heat *c*_P_ of EMF (Fig. 3B). It extends over the same temperature interval where the *s* parameter changes dramatically. We believe that the fast change of the specific heat should be attributed to freezing of loosely bound water. Noticeable spectral response of water is seen also in terahertz permittivity spectra (Supplementary Fig. 5), where the relaxational Debye term is needed to describe the low-frequency dispersion^35^: *ε*_D_ = Δ*ε*[1 + i*ωτ*]^−1^, where Δ*ε* is the relaxation strength, *τ* is the relaxation time and ω is the circular frequency. Thus, the Drude-like conductivity seen above 250–265 K (Fig. 1) in EMF can be ascribed to delocalized ions which flow in the liquid water phase. Similar spectral behaviors of conductivity, permittivity and specific heat are seen in CytC, though without anomaly in specific heat. No spectral or heat capacity signatures of loosely bound water are detected in BSA. Note that according to our thermogravimetry data (Supplementary Fig. 2), CytC and BSA samples contain smaller concentration of loosely bound water than EMF.

### Boson peak

**Figure 4:**
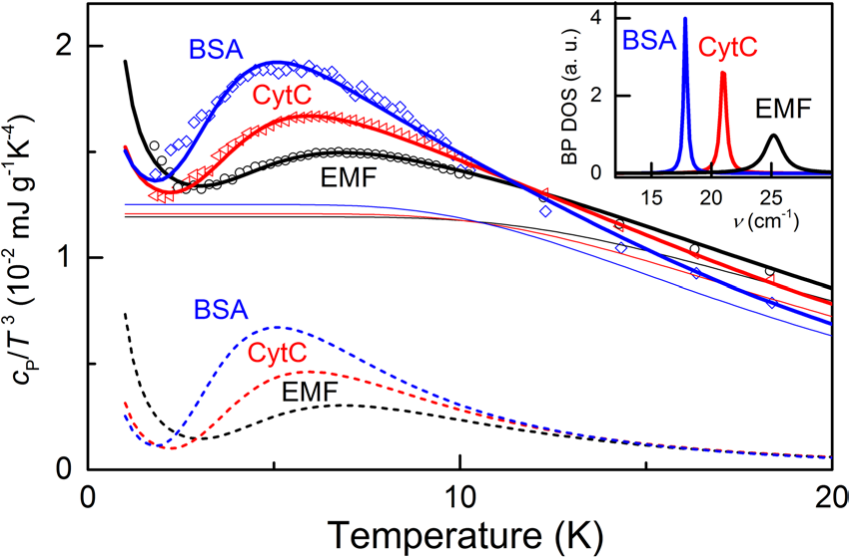
Dots – low-temperature specific heat of EMF, CytC and BSA, as indicated. Solid lines are fits to the data using expressions (3,4) (see Materials and Methods) with Debye and boson peak contributions shown separately with thin solid and dashed lines, respectively. Inset shows the boson peak density of states for the three samples modeled with Lorentzian lineshapes. Water contents in samples according to thermogravimetry measurements: EMF (~30%), CytC (~13%), BSA (~11%).

It is well-known that the disorder in solids results in the emergence of an excess contribution to the vibrational density of states (DOS) *g*(*ν*), relative to the Debye component *g*_D_(*ν*)~*ν* ^2^. This additional contribution, called boson peak, is observed by inelastic neutron or Raman scattering^36–41^ and reveals itself at THz frequencies as a maximum in the reduced DOS *g*_D_(*ν*)/ν^2^ and as a peak in the specific heat plotted as *c*_P_(*T*)/*T* ^3^ *vs T* ^42^. As is seen in Fig. 4, in all three materials, EMF, CytC and BSA, the *c*_P_(*T*)/*T* ^3^ data exhibit a clear maxima at around 5 K whose origin can be assigned to the boson peak phenomenon. We describe the low-temperature specific heat (solid lines in Fig. 4) by modeling the boson peak DOS *g*_BP_(*ν*) with a Lorentzian and taking into account regular Debye DOS term *g*_D_(*ν*) ∝ *ν* ^2^ (see Materials and Methods). The obtained Debye temperatures *Θ*_D_ (Table 1) should be considered as corresponding to certain characteristic vibration frequencies *ν*_D_ = k_B_ *θ*_D_/h (k_B_ – Boltzmann constant, h – Planck’s constant) of macromolecules within the systems that contribute to the specific heat. The observed boson peak positions fall in the range 0.5–0.7 THz (see Table 1) that includes the bands observed in crystals of chicken egg white lysozyme^43^. According to Perticaroli *et al*.^44^, the frequency of boson peak in protein correlates with the type of the dominant secondary structures: more rigid β-sheets in sample composition should lead to higher frequencies. This means that EMF should contain higher fraction of β-sheets than our reference proteins, CytC and BSA. This tendency matches the current view on the organization of cytochromes within periplasmic membrane of *Shewanella oneidensis*: they are trapped within large β-barrels making the structure more rigid^45^. The conductive filaments of *S. oneidensis* MR-1 are currently regarded as appendages of periplasmic membrane containing MtrB β-barrel porins in complex with OmcA and MtrA,C multiheme cytochromes^24^. Relatively more rigid behavior of CytC (if compared with BSA) can be attributed to the presence of heme molecule in the structure. In our optical THz studies (Supplementary Fig. 6) the boson peak feature revealed itself only in EMF, that is probably caused by higher level of hydration^46^.

We have conducted also the measurements of circular dichroism (CD) spectra of all three materials in the far ultraviolet. The studied suspensions were prepared directly from pellets utilized for dielectric spectroscopic measurements. Concentration of each material was 0.05 mg/ml. The resulting spectra are shown in the Supplementary Fig. 4. We can see that BSA and CytC yield canonical spectra typical for the medium with pH 7.0^47,48^. EMF demonstrates relatively low signal whose shape is very close to that in the spectra of pure β-sheet peptides^49^ and the published CD-spectra of OmcA protein in PBS buffer^25^. Thus, the CD data support the general picture yielded from boson peak parameters analysis, i.e., that EMF has the most rigid structure of all three studied materials. We have to note, however, that our value of the correlation length *ξ*_BP_ obtained for BSA is somewhat larger (3.8 ± 0.7 nm) than previously published by Sokolov *et al*. (2.20 nm)^44^. The reason of this discrepancy may come, e.g., from hydration level.

### Some speculations on the conductivity mechanisms in studied materials

What principle difference does exist in components of CytC and BSA? It seems that there are three important points here: (1) metallic ions in BSA, (2) heme molecules in CytC, (3) very close concentrations of water in the two samples, according to thermogravimetry. At the same time, the state of this water is principally different. This can be seen from the terahertz spectra: BSA does not demonstrate Debye-like relaxational dispersion at any temperature, while CytC does, importantly – together with the free carrier conductivity. It is hard to believe that the presence of sodium ions in BSA deprives such conductivity. It seems more probable that it is the presence of large conjugated aromatic heme system or bulk water phase that give CytC the ability to transfer free charges. The next question is then: what are these free charges that are delivered to CytC by heme? Are they ions or electrons? It is clear that heme of c-type is not a good ion donor, at least it is not “better” than any amino-acid with ionogenic side chain present in BSA. We thus come to the conclusion that the free carrier conductivity observed at room temperature in CytC is caused by heme and/or heme associated structures and relatively free water. The highest ionic concentration in CytC has iron (see Supplementary Table 1). The other ions have concentrations 2.5–100 times smaller. Since iron atoms are fixed within heme complexes, they cannot diffuse and cannot be effective ionic free carriers. The second position in the concentrations list holds sodium: 0.018% in CytC and 1.050% in BSA, that is by about six times higher. The differences in concentrations of other ions are not so dramatic. Concentration of quickly evaporating water in BSA is by about 2% smaller than in CytC, but this can be explained by higher sodium concentration (similar to what is observed in EMF). However, according to terahertz spectra, the behavior of water in CytC is completely different. Thus, we cannot exclude the case of free ionic conductivity in CytC, with the other, free electrons contribution still probable. The influence of water on the band structure of amorphous semiconductors with conjugated aromatics is well known for melanins^50,51^. We can thus assume that conjugation of bulk phase water and aromatics of heme groups lead to an activation of both, electronic and ionic free carrier conductivity, similar to what is observed in redox-sensitive aromatic systems like poly-semiquinones in melanins.

In its turn, EMF contains hemes (according to EPR data), metallic ions of high concentration (2.7% of potassium) and high quantity of bulk water (according to thermogravimetry and terahertz data). EMF reveals strong contribution of free carriers to the real part of conductivity, at room temperature and down to 245 K. Unlike in CytC, the presence of free carriers in EMF correlates with the existence of a knee-like feature in the specific heat behavior. It would be logical to assume that the observed feature is related to the freezing of weakly bond water and, thus, freezing of the ionic free carrier component.

## Conclusions

By using radio-frequency and terahertz spectroscopies, a number of characteristic dynamical temperature-frequency behaviors are observed in three different biological specimens, cytochrome C (CytC), bovine serum albumin (BSA) and *Shewanella oneidensis* MR-1 extracellular matrix and filaments (EMF). It is shown that the presence of aromatic heme groups in CytC and EMF does not significantly influence the free carrier conductivity observed in these two materials at temperatures below the freezing point of regular bulk water. A remarkable result of the study is the observation of loosely bound water phase in the samples, not only by means of traditional thermogravimetry technique but using terahertz spectroscopy that reveals Debye-like relaxation similar to that typically observed in the dielectric response of bulk water. Strict correlation is established between such relaxation and the presence of delocalized charge carriers in the EMF and CytC samples. The formal water content in the materials derived from thermogravimetry is found to carry no information on the state of this water: having significant amount of water (and sodium ions), BSA samples reveal no Debye-like relaxation and/or free carrier conductivity. Another essential finding is the correlation between the boson peak frequencies and circular dichroism data for the not so well studied material EMF, where higher frequencies of boson peak correspond to higher concentration of rigid beta-sheet structures and hemes.

Emerging dependencies are known to be universal for non-biological disordered systems, such as glasses, polymers or supercooled liquids. Our observations together with the origin of the dc conductivity in EMF and CytC need further studies with an account taken of the known concepts on the band conductivity in proteins and peptides^52–56^. We demonstrate that the use of broad-band dielectric spectroscopy opens up opportunities to utilize powerful technical and theoretical arsenals accumulated within the condensed matter physical community for the studies of diversity of biological phenomena and materials where charge and molecular dynamics is always crucial.

## Materials and Methods

### *Shewanella oneidensis* MR-1 strain origin

The strain *S. oneidensis* MR-1 was obtained by the Russian National Collection of Industrial Microorganisms, Scientific Center of Russian Federation - Research Institute for Genetics and Selection of Industrial Microorganism, from the collection of microorganisms of the Pasteur Institute (CIP106686, France). The strain *S. oneidensis* MR-1 has number B9861 in the Russian National Collection of Industrial Microorganisms.

### Cultivation of *Shewanella oneidensis* MR-1 strain

At the beginning of our research, we used a standard cultivation method, which involves the use of a minimal medium with lactate. However, later we compared the different media, both standard and proprietary, including rich media TSB etc. The comparison of the fuel cells operation using various media showed that the current obtained in the case of TSB medium and some other rich media does not inferior to the one obtained in case of minimal medium with lactate, while the duration of the stable fuel cell operation on a complete medium increases many times. The reproducibility and convergence of the results also increases during use of the complete media. Therefore, in the experiments purposed to obtain a biomass grown under conditions of electron acceptors deficiency we decided to utilize rich media. To generate conductive structures, *S. oneidensis* must be cultivated in a deficiency of available final electron acceptors, which are certainly present in a rich medium. However, a stable current generation in MFC in case of rich medium indicates an excess of the potential electron donors and a deficiency of the acceptors. A simple experiment supports this fact. It is impossible to grow *S. oneidensis* in rich medium (as well as in a minimal medium with lactate) under anaerobic conditions since the availability of electron acceptors is diminished. However, the growth can be observed after addition of an excess electron acceptor like fumarate or Fe^+3^ or introduction of the anode into the medium. In the case of current study, the *S. oneidensis* MR-1 strain was grown on Petri dishes on agarized Luria-Bertani medium (LBM) during 48 hours at 30 °C. For obtaining cell biomass, colonies from LBM were transferred into 100 mL of LB broth in a 750 mL flask and cultivated in aerobic conditions on a rotary shaker at 220 rpm at 30 °C for 18 hours. Cells were sedimented by centrifugation at 6000 g for 20 minutes, washed with sterile deionized water of Milli Q (Millipore, USA) under the same centrifugation conditions and resuspensed in 2 mL of deionized water. The biomass was transferred into MM synthetic medium^57^ with lactate concentration 4 g/L and then introduced into the anaerobic anodic chamber of microbial fuel cell. The anode and cathode of the fuel cell were made of stainless steel woven mesh (Russian steel grades TU-14-4-507-99) produced by Soyuznikhome (Russia). Anodic chamber volume was 250 ml. Cathodic chamber was aerobic. It contained 1x TAE buffer and had volume of 150 ml. The chambers were divided by reinforced nafion membrane (membrane thickness 160 microns) produced by Du Pont (USA). The cultivation of bacteria in anaerobic chamber was carried out until the current values reached 30-50 microamperes from cell (at a voltage of 0.4 volts with a load resistance of 8 kΩ). The density of cell culture in the medium reached 2–2.5 grams per liter.

### Isolation of *Shewanella oneidensis* MR-1 bacterial extracellular matrix and filaments (EMF)

The bacterial fuel cells that produced electrical currents exceeding 30 µA were used for isolation of EMF. These values fit well the published data^58^. The procedure included the following steps. The cell culture grown on an anode and washed with 100 mM phosphate buffer saline (рН 7.4) prepared from PBS tablets (Helicon, Russia) and pure water produced by Milli-Q integral water purification system (MERCK MILLIPORE, USA). After that the cells were concentrated by means of centrifugation (30 minutes, Beckman Coulter Avanti J-26 centrifuge, rotor JLA 8.1, speed 5500 rpm). The precipitate was collected into 50 ml falcon tubes. Every 5 ml of concentrated cells were diluted with 30 ml of Milli-Q water. The obtained suspension of cells was treated with intensive vortexing for avulsion of the filaments from the cells (maximal speed, Scientific Instruments Vortex-Genie 2, USA), for 10 minutes three times with two five-minutes breaks in water with ice between vortexing. After that 20 ml of Milli-Q water were added to each falcon tube to make 50 ml quantity. The falcon tubes were tightly shaked and then centrifuged for 15 min at 13000 g (Eppendorf 5810r centrifuge with FA-45–6–30 rotor). The supernatant was collected and treated with filtration through a 0.22 µm pore size Durapore membrane filter (MERCK MILLIPORE, USA). The precipitate was resuspended, vortexed for 5 minutes and re-centrifuged according to the procedure described above. The second supernatant was also filtrated trough a 0.22 µm Durapore membrane filter, mixed with the first supernatant and then cooled down to −80 °C in plates with large surface for further lyophilization. Small volume of filtered supernatant was collected for atomic-force microscopy study of the samples.

In accordance with current viewpoint of the leading laboratory in the field the conductive filaments produced by *S. oneidensis* MR-1 are an appendages of bacterial outer membrane^24^. The width of the appendages is much smaller than 0.22 μm, i.e. the size of the pore in the utilized filter. The membrane and appendages are supposed to contain multiheme cytochromes OmcA, MtrA and MtrC. And the last cytochrome being directly within membrane is surrounded by porin MtrB. The ‘porin-cytochrome’ model was also discussed in the article^59^. The appendages can be easily removed from the cells and isolated from the large cell debris impurities^22^.

### Lyophilization of EMF

The prepared and frozen extracellular matrix was lyophilized using the TFD 5503 freeze dryer (ilShin, South Korea). The lyophilization continued for 40 hours with gradual temperature increase from −80 °C to 36°C until the pressure of 0.010 mTorr was reached yielding amorphous light brown colored powder.

### The sources of reference proteins

Bovine serum albumin was supplied by Amresco, USA (code 0332). Bovine heart cytochrome C was supplied by Sigma-Aldrich, USA (code C3131).

### Sample pellets preparation

Amorphous powders of BSA, CytC and EMF were pressed into pellets using mold with diameter 10 mm. The pellets of BSA and CytC were obtained under pressure 10 atm. The pellets of EMF were obtained under pressure 5 atm.

### Water contents measurements

Water contents of all samples were estimated by means of dynamic thermogravimetry. We used the Netzsch STA 409 PC Luxx^®^ TGA-analyzer combined with quadruple mass-spectrometer 403 C Aëolos (70 keV). Heating rate was kept at 10 °C/min. During the heating process the sample was flushed with high purity argon (99.999%). Argon containing gaseous products entered the mass-spectrometer configured to track ion currents of water fragments with mass/charge (m/z) ratios [17]^−^ and [18]^−^ allowing to synchronize sample mass-loss with water allocation. In order to define the sample thermal stability we also tracked ion currents with m/z = [48]^−^ and [64]^−^ corresponding to molecular fragments SO^−^ and SO_2_^−^ respectively, thereby controlling the beginning of protein destruction. Simultaneity of [17]^−^ and [18]^−^ ion currents, as well as [48]^−^ and [64]^−^ allows us to state that we tracked H_2_O and SO_2_ molecules. The range of the applied temperature was 35–300 °C.

### The model to describe the boson peak phenomenon in specific heat data

We model the low-temperature dependences of the specific heat of our three samples with a sum of the Debye contribution and of the additional excess vibrational DOS due to boson peak that has a Lorentzian distribution:

3 $${g}_{BP}(\nu )\propto \frac{1}{{(\nu -{\nu }_{0})}^{2}+{\nu }_{\delta }^{2}}$$

The resulting specific heat is given by a standard expression^60^:

4 $${c}_{p} \sim \frac{d}{dT}[{\int }_{0}^{{\nu }_{D}}{g}_{D}\frac{\nu }{\exp (\frac{\nu }{T})-1}d\nu +{\int }_{0}^{{\nu }_{\max }}{g}_{BP}\frac{\nu }{\exp (\frac{\nu }{T})-1}d\nu ]$$

where *ν*_D_ is the Debye cutoff frequency related to the Debye temperature *Θ*_D_ via h*ν*_D_ = k_B_ *Θ*_D_, (with k_B_ and h being the Boltzmann and the Plancks’s constants, respectively), *v*_max_ > *v*_0_ + *v*_δ_, *v*_0_ is the frequency position of the boson peak and *v*_δ_ is its width. From the peak position we estimate the correlation length *ξ*, that is associated with the spatial scales of elastic constants fluctuations which produce the boson peak on a microscopic scale. Its value is usually defined as the ratio of the transverse sound velocity to the boson peak frequency as *ξ ≈* v_*s*_ */ν*_0_^61^. We calculate the values of *ξ* with the sound velocity estimated to be 2*10^5^ cm/s^62–64^.

### Dielectric measurements

For the low-frequency measurements plane-parallel samples of thickness 0.5–3 mm and diameter of about 1 cm were prepared. The dielectric measurements were performed using three spectrometers. At frequencies from ≈1 Hz to ≈1 MHz and temperatures 10 K to 300 K the dielectric response was measured in vacuum using a NOVOCONTROL Alpha AN High Performance Frequency Analyzer equipped with a He-flow cryostat JANIS ST-100. In the high radio-frequencies range (1–300 MHz, room temperatures) the sample impedance was measured by HP4191A impedance analyzer. Au-electrodes were applied to the faces of the samples plates. The contacts for applying the ac electric field were provided by silver wires fixed to the electrodes by a silver paste. In the terahertz and sub-terahertz ranges, the spectra of complex dielectric permittivity and optical conductivity were measured in a quasioptical (contactless) configuration using a spectrometer based on backward-wave oscillators operating at frequencies from 30 GHz to 1.5 THz and in the temperature interval from 2 K to 300 K. This is realized by measuring the spectra of complex (amplitude and phase) transmission coefficients of plane-parallel samples and subsequent evaluation of optical constants based on the Fresnel equations. The samples were cooled down to liquid helium temperatures in a helium-flow optical cryostat with Mylar windows. For infrared measurements, a standard Fourier-transform spectrometer Vertex 80 V was used to measure the spectra of reflection and transmission coefficients of the samples.

### DC conductivity measurements

Since most of equipment used for low-frequency dielectric measurements (impedance meters and frequency analyzers) operate in the 2-point scheme in which possible effects of electrical contacts to the sample cannot be reliably distinguished, it is always important to ensure that the contribution of the sample-contact interface is small enough as compared to the values corresponding to the bulk sample under study. In our experiments, we have verified the absence of noticeable contact effects in the EMF samples by comparing the low frequency ac conductivity (Fig. 1a) and the “true” four-contact dc conductivity measured in the classical linear geometry using Keithley 2400 for the test current and Keithley 6517 A as a precise voltmeter with high input resistance. The measurements were performed in the range 270–310 K and the temperature was regulated and stabilized in the home-made cryostat with SHI SRP-082 helium-free cryo-cooler and Lakeshore 340 temperature controller.

### Specific heat measurements

The specific heat data were collected by a relaxation technique in a Quantum Design physical property measurement system (PPMS) in the temperature range 300 K – 1.8 K. The samples of a rectangular shape with the mass ~10 mg were used.

### EPR measurements

Measurements were performed on Bruker Elexsys-500 (9 GHz) under 30 K with modulation frequency 100 KHz.

### Element analysis

Proteins were dissolved in concentrated sulfuric acid yielding final concentrations of 5–6 mg/ml. After that solutions were diluted 100 times by 2% nitric acid. Eventually, two dissolutions of every protein were prepared with 2% HNO_3_, 1% H_2_SO_4_ and 50–60 mkg/ml of protein (the rest is deionized water). These samples were analyzed by means of ICP-MS (iCAP Qc, Thermo Scientific). Measurements were performed on singly charged positive ions of the following isotopes: ^23^Na, ^24^Mg, ^25^Mg, ^26^Mg, ^39^K, ^44^Ca, ^56^Fe, ^57^Fe, ^63^Cu, ^65^Cu, ^66^Zn, ^68^Zn. Some of the measurements (^44^Ca, ^56^Fe) were performed using kinetic the energy discrimination regime to reduce possible polyatomic interferences such as ^12^C ^32^S or ^40^Ar ^16^O. To eliminate the impact of foreign contaminants, blank samples without proteins were also used. Solutions based on ICP-MS-68 Solution A were used for calibration. Concentration of every element was determined by averaging of all results evaluated from different isotopes and samples subtracting concentration of this element in blank samples. Detection limit was determined as mean of standard deviation of concentration of the corresponding element in blank samples multiplied by three. Measurements error was calculated as standard error of the mean multiplied by two.

### Circular Dichroism (CD)

To obtain CD spectra the sample pellets were collected after dielectric spectroscopy measurements and solved in standard 10 mM PBS buffer with pH 7.0. Final concentration for each material was 0.05 mg/ml. The spectra were obtained by means of Jasco J-1100 CD-spectrometer operating with the next parameters: spectral range 200–250 nm, data pitch 0.1 nm, scanning speed 50 nm/min, optical path length 10 mm. All measurements were performed at room temperature.

### Atomic-force microscopy of extracellular matrix and filaments before lyophilization

Scans are done in tapping mode on NTM-DT Smena Atomic Force Microscope system. Regular silicon cantilevers with resonant frequency 350 kHz were used.

### The note on reproducibility of results obtained on EMF

Five batches of EMF samples were prepared separately at different times. The dielectric spectral data in the range 10^−2^–10^6^ Hz was collected independently on two EMF samples in Prague (in the temperature range from 5 K to 300 K) and on the other two samples in Moscow (at room temperature), on different spectrometers by different operators. Corresponding spectral data obtained under the same conditions in different labs were identical. In parallel, samples from same batches were used in experiments on heat capacity (Dresden), elements’ and water concentration analysis (Moscow), EPR and circular dichroism (Moscow). Heat capacity temperature measurement was performed one time on the EMF sample sent to Dresden lab from Prague where samples of the same batch were studied in radio-frequency experiments. Thermogravimetry measurements were performed two times on the samples from batches studied in Moscow. Element analysis was performed one time on the sample from the batch studied in Moscow. Circular dichroism experiments were performed in Moscow two times: on the samples returned from Prague and on the sample from the batch studied in the radio-frequency range in Moscow, both yielding same results. Terahertz range measurements were performed only in Moscow on the samples from three batches. All of them provided same results.

### The note on reproducibility of results obtained on BSA and CytC

Unlike EMF, BSA and CytC were obtained commercially. Each material was measured twice in Moscow, and three times in Prague in the radio-frequency range at one level of hydration estimated by thermogravimetry as ~13% for CytC and ~11% for BSA. In the terahertz frequency range we measured both materials five times on the samples with different hydration levels, corresponding data not presented in this paper. The heat capacity measurements on both samples, CytC and BSA, were performed one time, at the level of hydration ~13% for CytC and ~11% for BSA, as in case of other results presented in this paper. Circular dichroism spectra were measured one time and agreed with the literature data. We did not perform EPR spectra measurements for CytC and BSA.

### Data availability

The data that support the findings of this study are available from the authors on reasonable request. Please, see author contributions for specific data sets.

## Electronic supplementary material

Supplementary information
