# Supplementary material for: Observation of dielectric universalities in albumin, cytochrome C and Shewanella oneidensis MR-1 extracellular matrix
Source: Sci Rep. 2017 Nov 16;7:15731. doi: 10.1038/s41598-017-15693-y (PMC5691187; doi:10.1038/s41598-017-15693-y)
Supplement: Supplementary file 1 — Supplementary information [file 41598_2017_15693_MOESM1_ESM.docx]

**Supplementary information**

**Observation of dielectric universalities in albumin, cytochrome C
and *Shewanella oneidensis* MR-1 extracellular matrix**

K.A.Motovilov, M.Savinov, E.S.Zhukova, A.A.Pronin, Z.V.Gagkaeva, V.Grinenko, K.V. Sidoruk, T.A.Voeikova, P.Yu.Barzilovich, A.K.Grebenko, S.V.Lisovskii, V.I.Torgashev, P.Bednyakov, J.Pokorný, M.Dressel, and B.P. Gorshunov

Email: k.a.motovilov@gmail.com*,* bpgorshunov@gmail.com


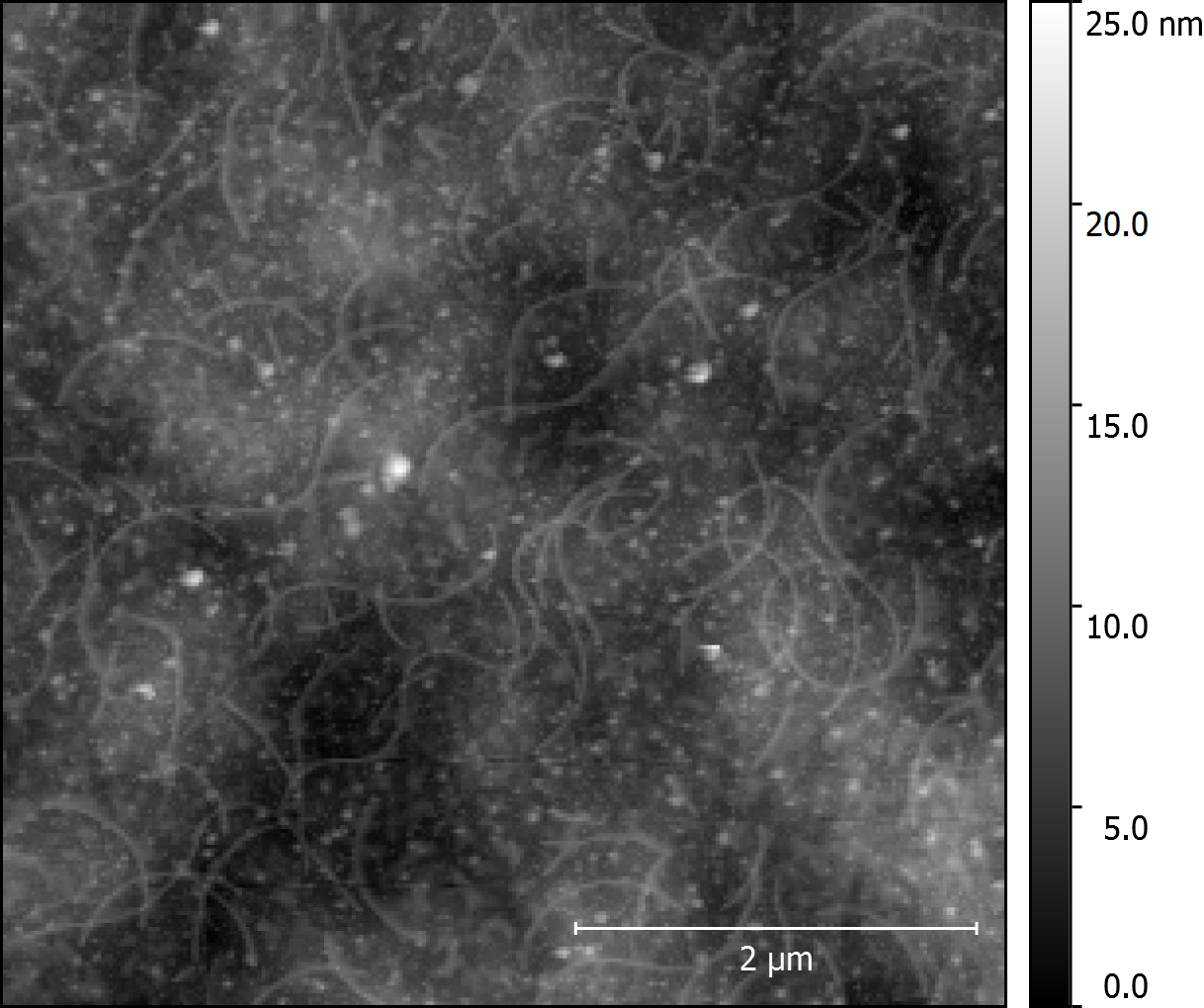


**Supplementary Figure 1.** AFM image of the filaments in the suspension utilized for preparation of EMF samples.

**
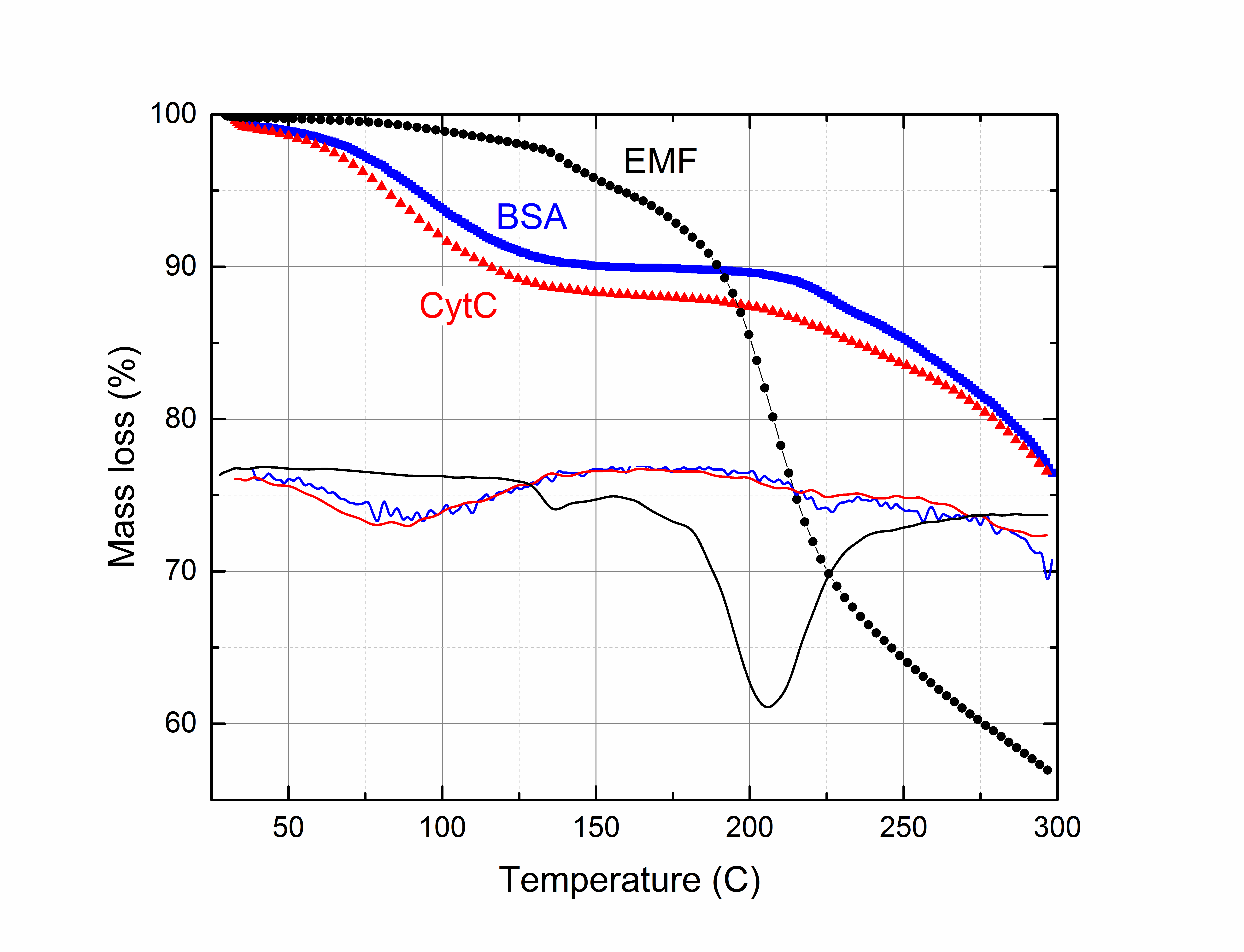
**

**Supplementary Figure 2.** Thermogravimetry data of studied materials (dots) demonstrates that the extracellular matrix and filaments (EMF) sample behaves in a different way compared to cytochrome C (CytC) and bovine serum albumin (BSA) proteins. Solid lines with corresponding colors show first derivatives. The main water loss is seen at temperatures below 150 °C in the case of reference materials (BSA, CytC). At this temperature, EMF loses only about 4% of mass, but total water loss reaches about 25-30% at 200-220 °C, where sulfur containing gases start to release from the samples. We believe that such behavior of EMF has ebullioscopic origin, since this material contains relatively high concentrations of ions (for details see supplementary Table 1 with element analysis data). Slight shift towards higher temperatures of characteristic mass loss observed in BSA (if compared with CytC) also may arise from higher concentration of metallic ions.

**
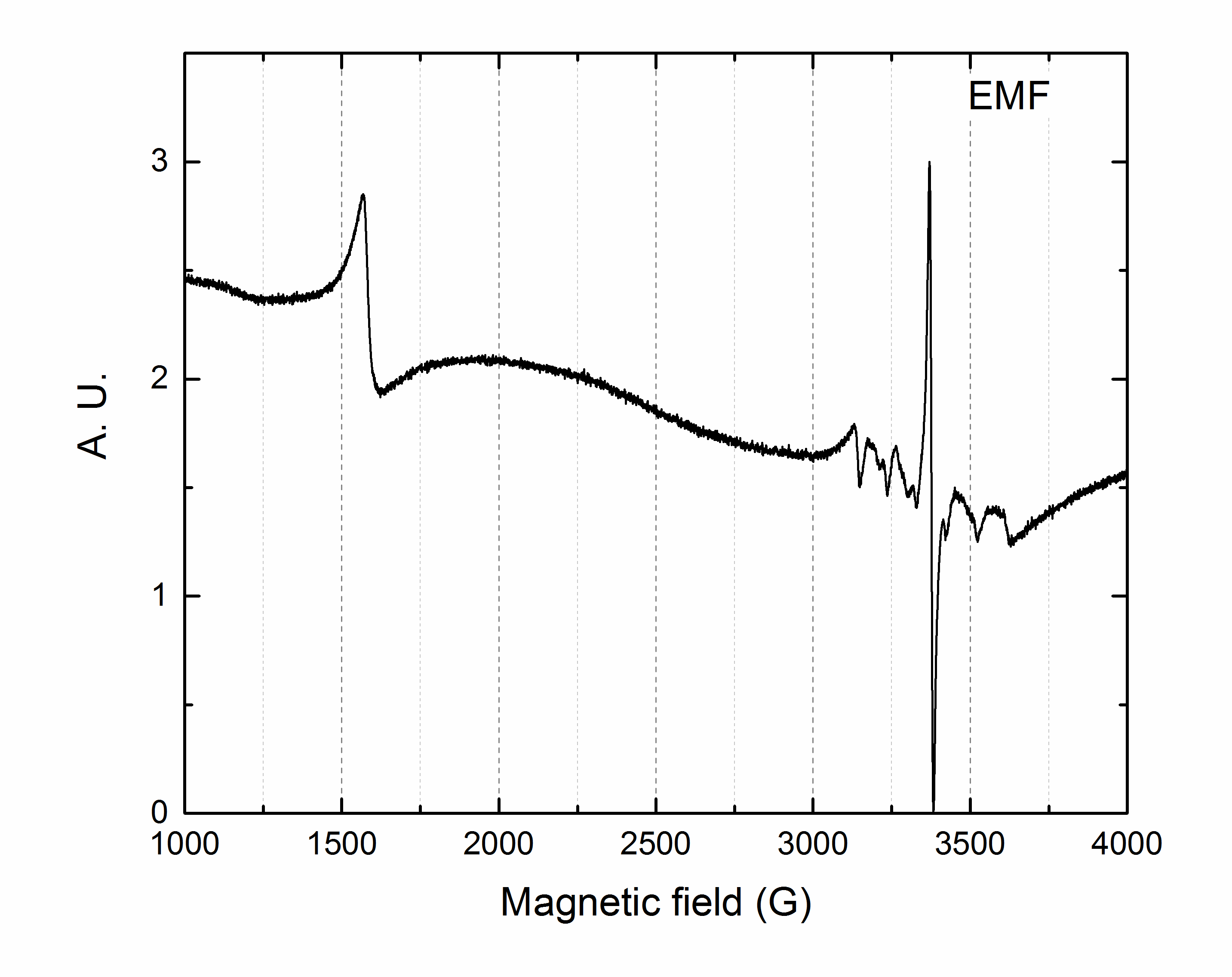
**

**Supplementary Fig. 3.** EPR spectra of EMF at 30 K. It should be compared with the published data on MtrA^1^, MtrC^2^ and OmcA^3^ multiheme cytochromes of *Shewanella oneidensis* MR-1. The spectrum reveal spin and oxidation states of iron atoms and can help to distinguish MtrX and OmcY (X=C,F,A, Y=A,B) species to estimate their relative fractions in the mixture. Our data obtained at 30 K demonstrate broad peak at 1900-2600 G corresponding to the mixture of various Fe^2+^ ions in intermediate spin state and weak signal with the g factor g=6 that stands for high spin Fe^3+^ ions. Also, the data show high amount of iron with g=4.3. Correlation with previously published data allows us to suggest that the main part of EPR-active substances inside EMF contains large amounts of MtrC and OmcA species in almost equal quantities as it should be in *Shewanella oneidensis* MR-1 nanofilaments involved in charge transfer.


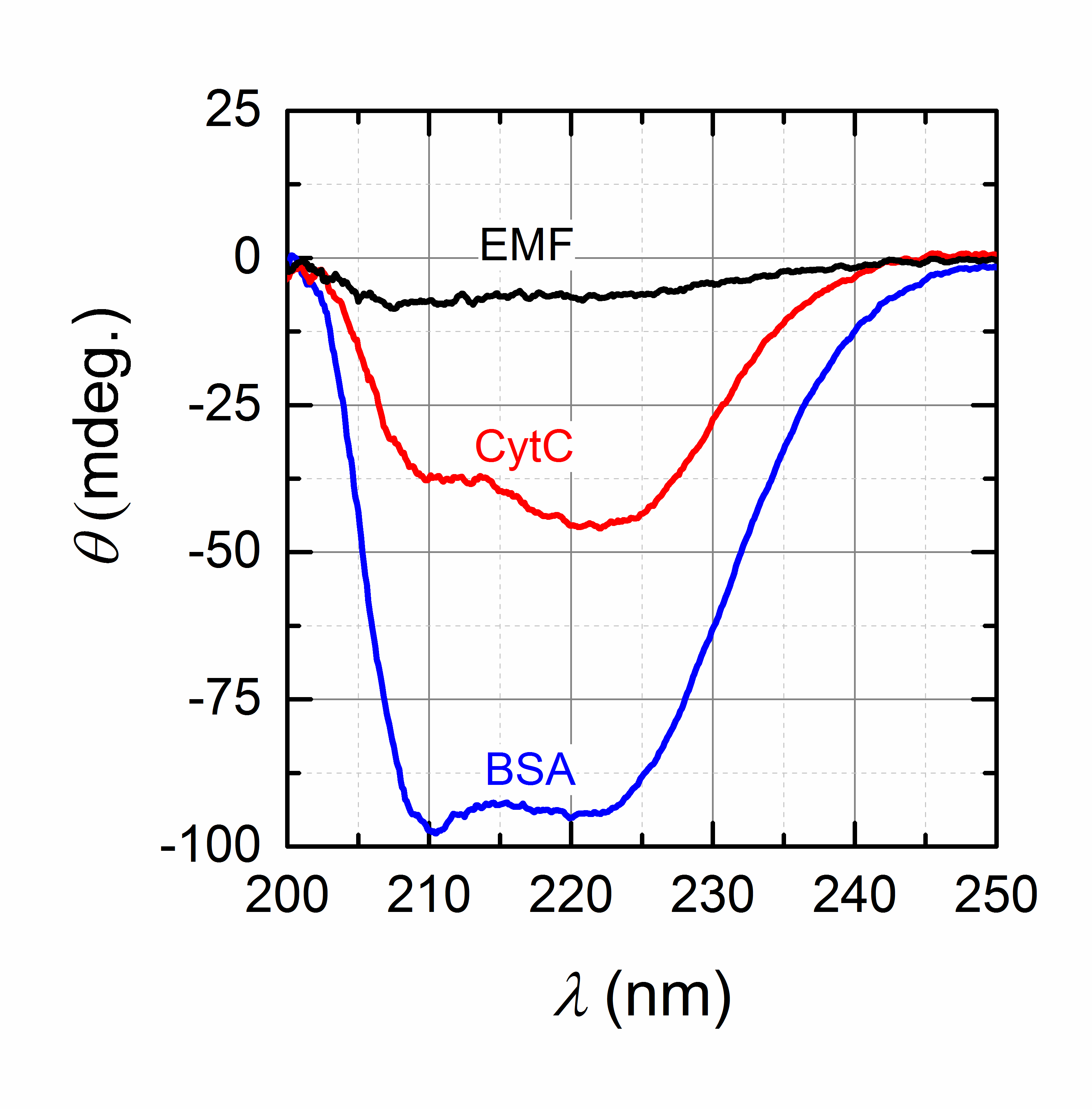


**Supplementary Fig. 4.** Circular dichroism spectra of BSA (blue line), CytC (red line) and EMF (black line). Optical path length is 10 mm. Concentration of each sample is 0.05 mg/ml.


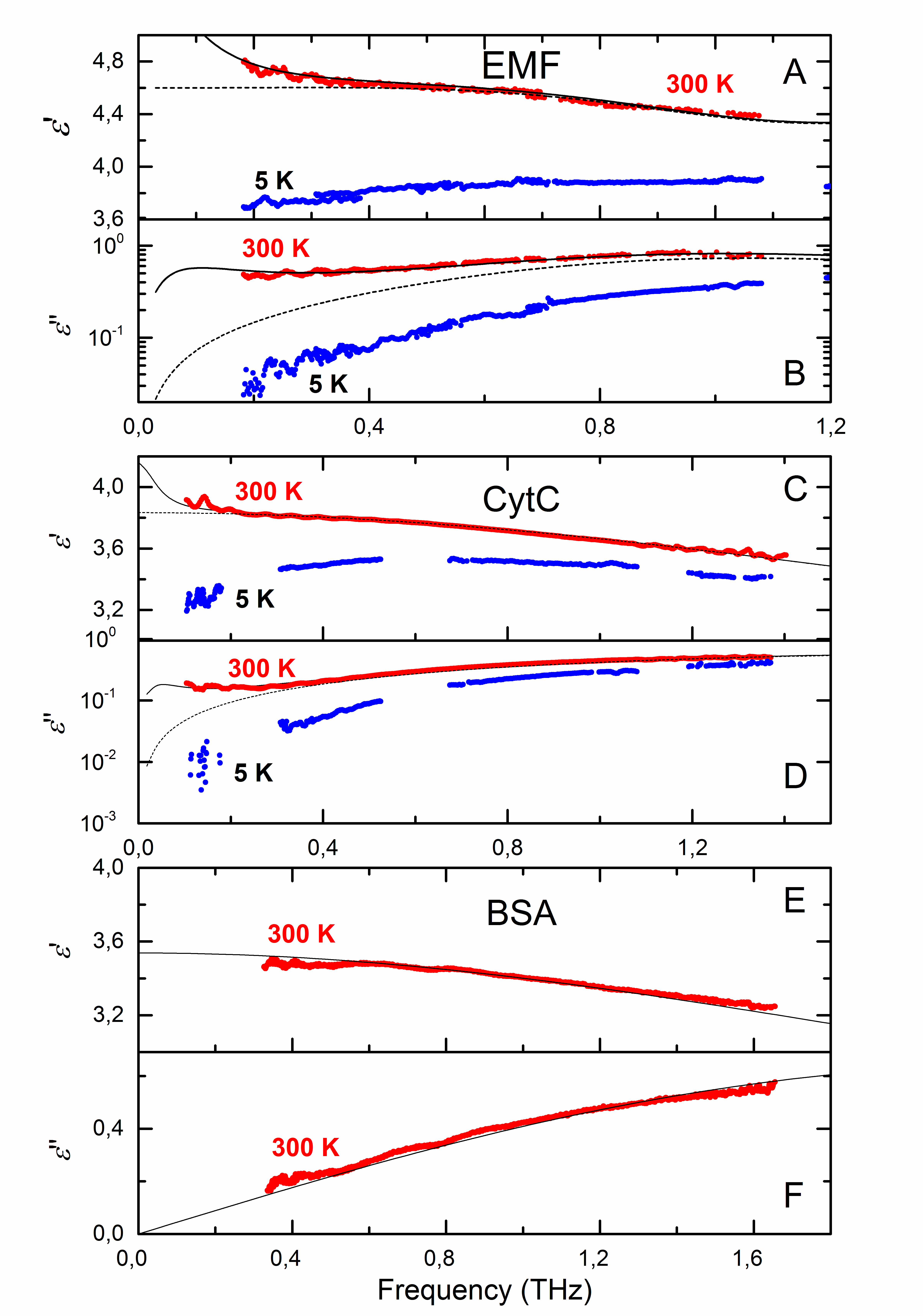


**Supplementary Figure 5.** Terahertz spectra of real (A, C, E) and imaginary (B, D, F) permittivity of EMF (A, B), CytC (C, D) and BSA (E, F), obtained at room temperature (red) and 5 K (blue). Solid black lines show fits with Lorentzian describing higher-frequency dispersion and Debye-type relaxation describing lower-frequency contribution due to bulk water phase. Dashed black lines show same fits but without the Debye term (see text). Absence of Debye-like contribution in BSA even at room temperature indicates absence of bulk water phase. According to thermogravimetry data, water content in EMF was about 30%, in CytC – 12-14%, in BSA – 10-12% depending on particular sample. The data on supplementary figures 2, 5, 6 were obtained on the same samples and at the same levels of hydration.


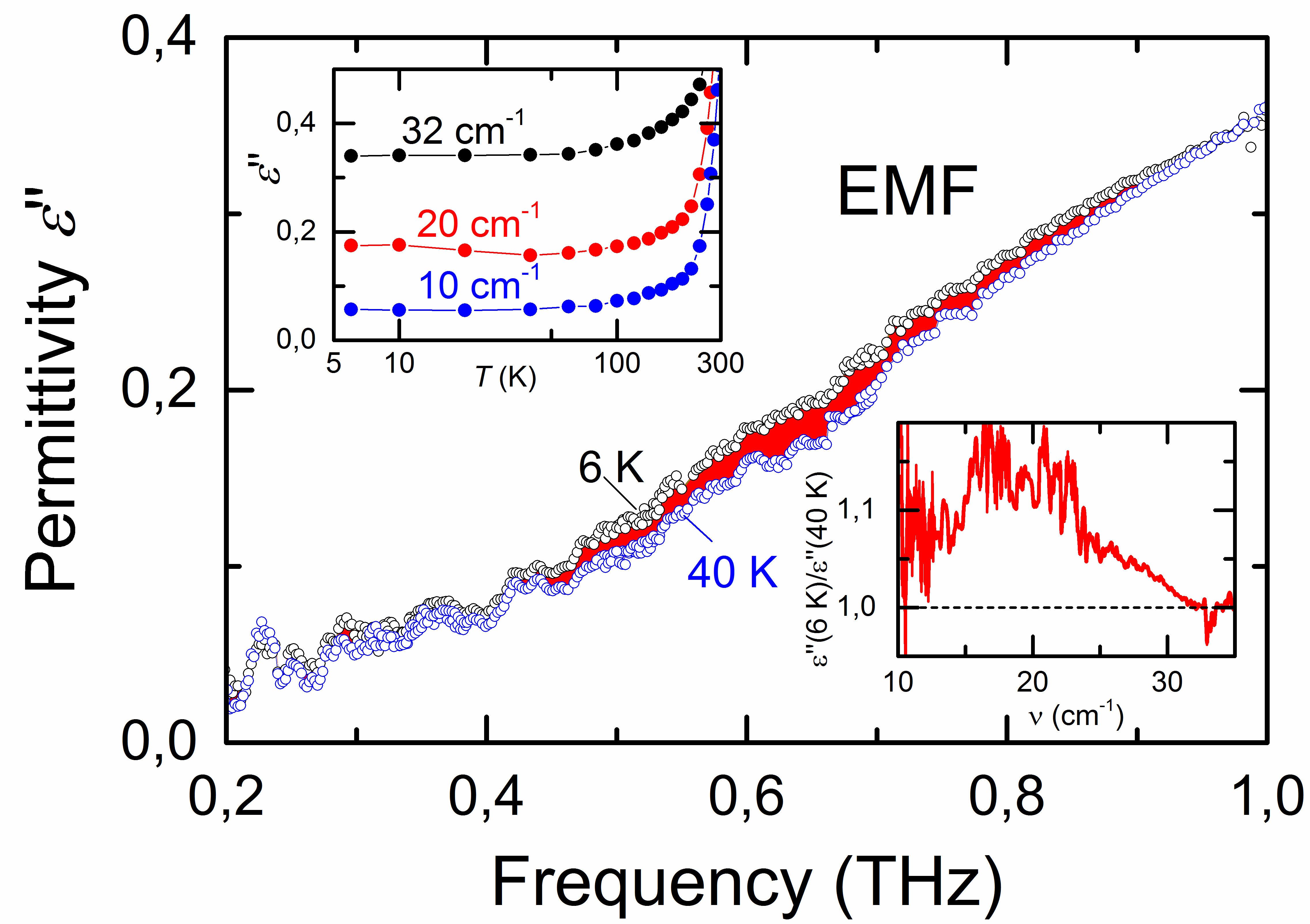


**Supplementary Figure 6.** Terahertz spectra of imaginary permittivity of extracellular matrix and filaments (EMF) measured at 6 K and 40 K. Filled area between the spectra marks absorption caused by the boson peak that is activated in the THz spectra at the lowest temperatures due to breaking of the selection rules, and that causes an increase of loss at a frequency of 0.6 THz (upper inset). Lower inset shows the result of division of the two spectra – the boson peak. Water content of the studied sample was about 30%.

**Supplementary Table 1.** Mass concentrations of various metals in the studied samples of cytochrome C (CytC), bovine serum albumin (BSA) and extracellular matrix and filaments (EMF), measured by means of mass-spectrometry. Concentrated sulphuric acid was used to dissolve the samples. Concentration of every element was determined by averaging of all results evaluated from different isotopes and samples subtracting concentration of this element in blank samples. Detection limit was determined as mean of standard deviation of concentration of the corresponding element in blank samples multiplied by three. Measurements error was calculated as standard error of the mean multiplied by two. More experimental details are presented in Material and Methods section.

| **Element** | **CytC, %** | | **BSA, %** | | **EMF, %** | | **H_2_SO_4_ - control, 10^-4^%** |
| --- | --- | --- | --- | --- | --- | --- | --- |
| Na | 0.018 | ±0.006 | 1.050 | ±0.003 | 0.33 | ±0.02 | 3.4 |
| Mg | 0.0088 | ±0.0002 | 0.0041 | ±0.0002 | 0.100 | ±0.003 | 4.9 |
| K | 0.007 | ±0.007 | 0.052 | ±0.004 | 2.7 | ±0.2 | 1.7 |
| Ca | 0.004 | ±0.001 | 0.002 | ±0.002 | 0.007 | ±0.001 | 1.8 |
| Fe | 0.41 | ±0.03 | 0.002 | ±0.002 | 0.063 | ±0.003 | 2.3 |
| Cu | 0.003 | ±0.004 | 0.002 | ±0.005 | 0.002 | ±0.004 | 9.9 |
| Zn | 0.002 | ±0.005 | 0.002 | ±0.006 | 0.04 | ±0.02 | 6.1 |

**REFERENCES**

1. Pitts, K. E. *et al.* Characterization of the Shewanella oneidensis MR-1 Decaheme Cytochrome MtrA. *J. Biol. Chem.* **278,** 27758–27765 (2003).

2. Hartshorne, R. S. *et al.* Characterization of Shewanella oneidensis MtrC: a cell-surface decaheme cytochrome involved in respiratory electron transport to extracellular electron acceptors. *JBIC J. Biol. Inorg. Chem.* **12,** 1083–1094 (2007).

3. Bodemer, G. J., Antholine, W. A., Basova, L. V., Saffarini, D. & Pacheco, A. A. The effect of detergents and lipids on the properties of the outer-membrane protein OmcA from Shewanella oneidensis. *JBIC J. Biol. Inorg. Chem.* **15,** 749–758 (2010).
